# Resveratrol Inhibits ROS-Promoted Activation and Glycolysis of Pancreatic Stellate Cells via Suppression of miR-21

**DOI:** 10.1155/2018/1346958

**Published:** 2018-04-26

**Authors:** Bin Yan, Liang Cheng, Zhengdong Jiang, Ke Chen, Cancan Zhou, Liankang Sun, Junyu Cao, Weikun Qian, Jie Li, Tao Shan, Jianjun Lei, Qingyong Ma, Jiguang Ma

**Affiliations:** ^1^Department of Hepatobiliary Surgery, First Affiliated Hospital of Xi'an Jiaotong University, Xi'an 710061, China; ^2^Department of General Surgery, Second Affiliated Hospital of Xi'an Jiaotong University, Xi'an 710004, China; ^3^Department of Anesthesiology, First Affiliated Hospital of Xi'an Jiaotong University, Xi'an 710061, China

## Abstract

Activation of pancreatic stellate cells (PSCs) initiates pancreatic fibrosis in chronic pancreatitis and furnishes a niche that enhances the malignancy of pancreatic cancer cells (PCCs) in pancreatic ductal adenocarcinoma (PDAC). Resveratrol (RSV), a natural polyphenol, exhibits potent antioxidant and anticancer effects. However, whether and how RSV influences the biological properties of activated PSCs and the effects of these changes on tumor remain unknown. In the present study, we found that RSV impeded hydrogen peroxide-driven reactive oxygen species- (ROS-) induced activation, invasion, migration, and glycolysis of PSCs. In addition, miR-21 expression in activated PSCs was downregulated after RSV treatment, whereas the PTEN protein level increased. miR-21 silencing attenuated ROS-induced activation, invasion, migration, and glycolysis of PSCs, whereas the overexpression of miR-21 rescued the responses of PSCs treated with RSV. Moreover, RSV or N-acetyl-L-cysteine (NAC) administration or miR-21 knockdown in PSCs reduced the invasion and migration of PCCs in coculture, and the effects of RSV were partly reversed by miR-21 upregulation. Collectively, RSV inhibits PCC invasion and migration through suppression of ROS/miR-21-mediated activation and glycolysis in PSCs. Therefore, targeting miR-21-mediated glycolysis by RSV in tumor stroma may serve as a new strategy for clinical PDAC prevention or treatment.

## 1. Introduction

With a 7% five-year survival rate and more than 43,000 estimated deaths per year in the United States, pancreatic ductal adenocarcinoma (PDAC) is one of the most lethal cancers and causes serious public health issues and cancer burden [1]. PDAC is expected to become the second leading cancer diagnosis in the US by 2030, surpassing breast, prostate, and colorectal cancers [2]. Aggressive growth and metastasis, lack of early diagnosis, low rates of curative resection, and poor responses to radiation and chemotherapy characterize the dismal prognosis and treatment of PDAC. Modified combination regimens and novel agents are urgently needed, and the mechanisms of pharmacological therapies for PDAC remain to be further elucidated.

Current therapies focus chiefly on epithelial cancer cells, which contribute to the rapid proliferation and malignancy of the tumor. However, the tumor microenvironment, which is formed from a bulk of desmoplastic stroma comprising immune cells, inflammatory cells, endothelial cells, extracellular matrix, and, predominantly, pancreatic stellate cells (PSCs), plays an important role in tumor invasion and metastasis [3–5]. PSC activation, which is characterized by a decrease in vitamin A-containing lipid droplets and increased expression of *α*-SMA and collagen-I, is pivotal in the development of pancreatic fibrosis and the malignant behavior of PDAC [6, 7]. PSCs are activated by TGF-*β* as well as cellular reactive oxygen species (ROS), and cancer cells can secrete hydrogen peroxide (H_2_O_2_), which triggers oxidative stress in adjacent PSCs [8, 9]. The interaction between PSCs (cancer-associated fibroblasts) and tumor cells is mediated by diverse secreted soluble factors such as extracellular matrix proteins, cytokines, and integrins [10], and disrupting this connection may provide novel approaches to cancer therapy.

According to the widely accepted Warburg effect, tumor cells favor a metabolic shift toward glycolysis even under aerobic conditions. Remarkably, cancer-associated fibroblasts, particularly PSCs, are commonly “corrupted” and tend to increase glycolysis and autophagy to function as factories to convert glucose to lactate and then transfer abundant metabolites to cancer cells. This two-compartment model is defined as the “reverse Warburg effect” [11, 12]. miR-21, a small single-stranded noncoding RNA classified as an oncogenic microRNA that can regulate gene expression, is aberrantly expressed in the majority of human cancers such as pancreatic cancer [13], cervical cancer [14], and breast cancer [15]. High expression of miR-21 in the tumor stroma promotes tumor progression [16–18]. Inhibition of miR-21 induces apoptosis and cell cycle arrest and enhances the chemotherapeutic sensitivity of tumors by positively modulating PTEN, PDCD4, and BCL-2 and other target genes [15, 19]. Glycolysis is also impeded by downregulation of miR-21 in bladder cancer cells [20]. However, whether aberrant miR-21 expression promotes glycolysis and the invasiveness of PSCs remains unclear.

Resveratrol (trans-3,4′,5-trihydroxystilbene, RSV), a natural polyphenol detected in grapes, berries, and peanuts, has a wide spectrum of pharmacological properties, such as antioxidant [21], anti-inflammation [22], and antitumor effects [23]. RSV inhibits tumor growth, invasion, and epithelial-mesenchymal transition [24] and enhances chemosensitivity [25]. RSV can impede tumor cell proliferation by reducing the phosphorylation of PI3K, Akt, ERK, FOXO3a (Ser253), and FOXO1 (Ser256). Furthermore, RSV induces apoptosis and cell cycle arrest in tumor cells by enhancing expression of p21, p27, Bim, and cleaved caspase-3 and by inhibiting the expression of cyclin D1 [26]. There are other typical genes and pathways regulated by RSV such as NF-*κ*B, SIRT1-regulated pathway, and Wnt signaling pathway [27]. It is also worth noting that RSV may exert anti-inflammation and antitumor effects by targeting microRNAs [28]. Glycolysis of tumor cells is disrupted by RSV via the modulation of glucose consumption and glycolytic enzymes such as hexokinase 2 [29, 30]. However, whether and how RSV influences the invasion, migration, or glycolysis of activated PSCs and the effects of these changes on tumor biological properties remain unknown.

In this study, we demonstrated that RSV may inhibit ROS-promoted activation, invasion, and glycolysis of PSCs via suppression of miR-21. More importantly, impeded activation and lactate secretion of PSCs attenuated pancreatic cancer cell invasion and migration in coculture.

## 2. Materials and Methods

### 2.1. Reagents

Resveratrol (>99% pure), MTT (3-(4,5-dimethyl-2-thiazolyl)-2,5-diphenyl-2-H-tetrazolium bromide), and NAC (N-acetyl-L-cysteine) were purchased from Sigma-Aldrich (St. Louis, MO, USA). Analytical grade 30% H_2_O_2_ was obtained from Sinopharm Chemical Reagent Co., Ltd. (Shanghai, China). 2′-7′-Dichloro-dihydro-fluorescein diacetate (DCFH-DA) was obtained from Beyotime Institute of Biotechnology (Haimen, China).

### 2.2. Cell Lines and Cell Culture

Human PSCs were isolated from normal pancreatic tissue removed from patients undergoing liver transplantation. These tissues were obtained from the Department of Hepatobiliary Surgery at the First Affiliated Hospital of Xi'an Jiaotong University. PSCs were isolated and cultured according to methods described in previous study [31]. PSCs between passages 1 and 4 after isolation were used in our experiments. The human pancreatic cancer cell line Panc-1 was purchased from the Type Culture Collection of the Chinese Academy of Sciences (Shanghai, China) and cultured in Dulbecco's modified Eagle medium (DMEM) containing 10% FBS (HyClone, Logan, UT, USA), 100 *μ*g/mL ampicillin, and 100 *μ*g/mL streptomycin at 37°C with 5% CO_2_ and 95% air. The study was conducted following the Declaration of Helsinki, and the protocol and consent forms were approved by the relevant ethical committee of the First Affiliated Hospital of Xi'an Jiaotong University, China.

### 2.3. Cell Viability Assay

PSCs were plated into 96-well plates at a density of 6000 cells/well and treated with various concentrations (0, 12.5, 25, 50, 100, and 200 *μ*M) of RSV for 24, 48, and 72 h and different concentrations (0, 12.5, 25, 37.5, 50, 62.5, 75, 87.5, 100, 150, 200, and 300 *μ*M) of H_2_O_2_ for 24 h. Cell viability was assessed by the MTT assay. Ten microliters of 5 mg/mL MTT was added to each well after removing the media and incubated at 37°C for 4 h. Then, 100 *μ*L of DMSO was added to each well, and the optical density (OD) was measured at 490 nm on a multifunction microplate reader (POLARstar OPTIMA; BMG, Offenburg, Germany). The proliferation inhibition rate was calculated according to the following equation: proliferation inhibition rate = (1 − OD sample/OD control) × 100%.

### 2.4. Transfection of miRNA Inhibitor and Mimics

Loss-of-function and gain-of-function approaches were performed using the miR-21 inhibitor, negative inhibitor, miR-21 mimics, and negative mimics, which were purchased from GenePharma (Shanghai, China), and the sequences are provided in Supplementary Materials Table S1. The miRNA inhibitor and mimics were transfected into PSCs using Lipofectamine 2000 according to the manufacturer's instructions, and 8 h after transfection, the medium was replaced with fresh Dulbecco's modified Eagle medium: Nutrient Mixture F-12 (DMEM-F12) and cells were prepared for further experiments.

### 2.5. Western Blot Analysis

Western blot experiments have been described previously [25]. The antibodies used in this study against glucose transporter 1 (Glut1), hexokinase 2 (HK2), pyruvate kinase M2 (PKM2), and lactate dehydrogenase A (LDHA) were from Proteintech Group (Chicago, IL). The primary antibody against *α*-SMA was from Sigma-Aldrich. The primary antibody against PTEN was from Abcam (Cambridge, MA, UK).

### 2.6. Real-Time PCR

Total RNA was extracted using the Fastgen1000 RNA isolation system (Fastgen, Shanghai, China) according to the manufacturer's protocol. Total RNA was reverse-transcribed into cDNA using the PrimeScript RT reagent kit (TaKaRa, Dalian, China). A Bulge-Loop miRNA qRT-PCR primer set specific for miR-21 and U6 was obtained from RiboBio (Guangzhou, China). Real-time PCR was conducted using the CFX Manager 2.1 fluorescent quantitative PCR kit (Bio-Rad Laboratories, Hercules, CA, USA) under the following conditions: 10 min at 95°C and 40 cycles of 95°C for 2 sec, 60°C for 20 sec, and 70°C for 10 sec.

### 2.7. Immunofluorescence Staining

Cells were fixed in 4% formaldehyde diluted in phosphate-buffered saline (PBS) for 15 min, permeabilized with 0.3% Triton X-100, treated with blocking buffer (5% BSA in PBS), and then incubated overnight with the primary antibody at 4°C. The cells were then incubated with the Red-conjugated secondary antibody from Jackson ImmunoResearch Laboratories (West Grove, PA, USA) for 1 h at room temperature. Slides were mounted and examined using a Zeiss Instruments confocal microscope.

### 2.8. Measurement of Intracellular ROS

The level of intracellular ROS was measured using the ROS assay kit. In brief, after removing the media and washing the wells twice with PBS, 10 *μ*M of DCFH-DA was added to each well. Then, the cells were incubated in the dark at 37°C for 30 min. After washing twice with PBS and trypsinization, the cells were collected and analyzed immediately by flow cytometry using a FACSCalibur (BD Biosciences, San Diego, CA, USA) instrument.

### 2.9. Measurement of Lactate Production

Medium from cultured cells was collected, and a lactic acid assay kit (Nanjing Jiancheng Bioengineering Institute, Nanjing, China) was used to determine lactate production. Lactate concentration was measured by an enzymatic assay, which generates a colorimetric (530 nm) product, proportional to the lactate present. All procedures were performed according to the manufacturer's instructions.

### 2.10. Oil Red O Staining

After washing with cold PBS, cells were fixed in 4% paraformaldehyde for 15 min at room temperature. Cells were washed again with PBS and stained using filtered Oil Red O solution (Sigma, St. Louis, MO, USA) at 60°C for 30 min. Subsequently, hematoxylin was used to stain the cell nuclei. Intracellular lipid accumulation was examined through a microscope (Nikon Instruments Inc.).

### 2.11. Modified Wound Healing Assay

Cell migratory ability was detected by a modified wound healing assay. To assess the migratory abilities of PSCs, PSCs (5.0 × 10^5^ cells/2.5 mL) were seeded into 6-well plates using DME/F12. To evaluate the migratory abilities of pancreatic cancer cells (PCCs) under coculture, Panc-1 (1.0 × 10^6^ cells/2.75 mL) and PSCs (5.0 × 10^5^ cells/2 mL) were seeded into the basolateral and apical sides of Millicell hanging cell culture inserts (pore size 0.4 *μ*m) in 6-well plates, respectively, using a mixed culture medium (DMEM : DME/F12 = 1 : 1). After the cells grew to 90–100% confluency, a sterile pipette tip was used to produce a wound line between the cells in the plate. Cellular debris was removed by washing with PBS, and the cells were then allowed to migrate for 24 h. Images were captured at time 0 and 24 h post wounding under a Nikon Diaphot TMD inverted microscope. The relative distance traveled by the leading edge from 0 to 24 h was assessed using the Photoshop software (*n* = 3).

### 2.12. Modified Transwell Matrigel Invasion Assay

Modified Transwell Matrigel invasion assays were performed in Transwell chambers. The 8.0 *μ*m pore inserts were coated with 25 *μ*L of Matrigel. To assess the invasive abilities of PSCs, PSC suspensions (5 × 10^4^) were added to the upper chambers in DME/F12 containing 1% FBS. Simultaneously, 500 *μ*L of DME/F12 containing 10% FBS was placed in the lower chambers. To evaluate the invasive abilities of PCCs under coculture, Panc-1 suspensions (5 × 10^4^) were added to the upper chambers in DMEM/DME/F12 (1 : 1) containing 1% FBS. Simultaneously, 500 *μ*L of DMEM/DME/F12 (1 : 1) containing 10% FBS was placed in the lower chambers with different groups of PSCs (5 × 10^4^). The cells were allowed to migrate for 48 h at 37°C. The noninvading cells were removed from the upper surface by scraping with a wet cotton swab. After rinsing with PBS, the filter was fixed and stained with crystal violet. Invasion ability was determined by counting the stained cells on the bottom surface of each membrane in 10 random fields, and images were captured at ×200 magnification (*n* = 3).

### 2.13. Statistical Analysis

Each experiment was independently performed at least three times. Data are presented as means ± standard deviation. Differences were evaluated using Student's *t*-test, with *p* < 0.05 considered statistically significant.

## 3. Results

### 3.1. RSV Inhibits H_2_O_2_-Promoted PSC Activation, Migration, and Invasion

**Figure 1:**
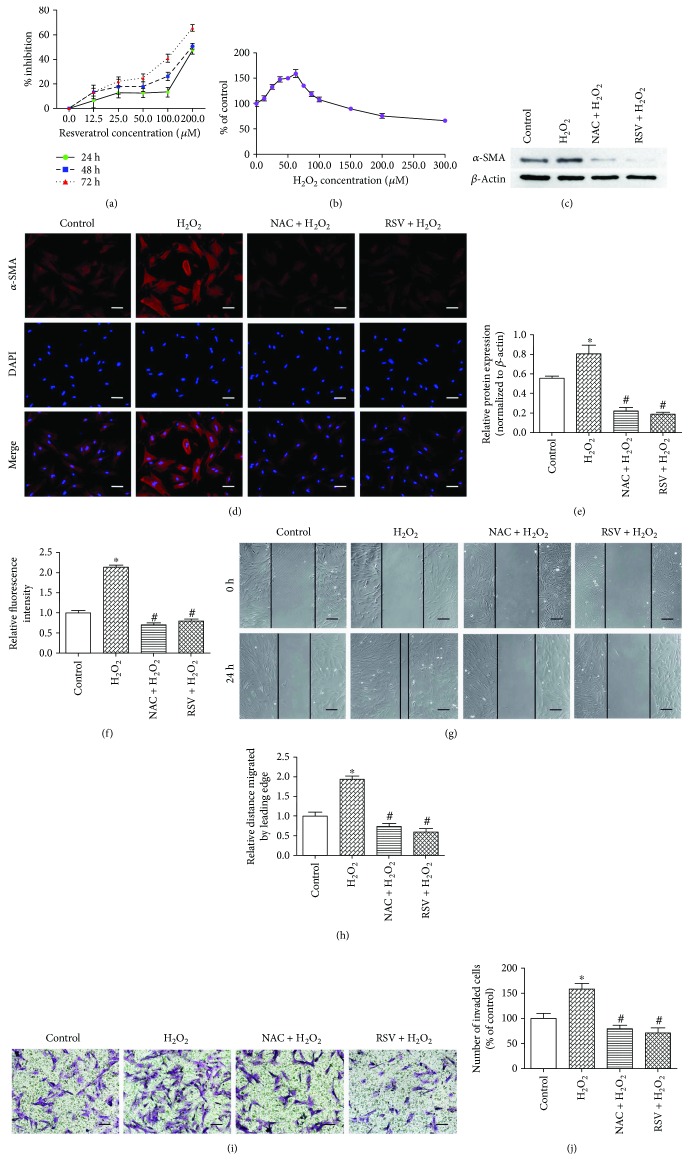
Resveratrol (RSV) inhibits hydrogen peroxide (H_2_O_2_)-promoted pancreatic stellate cell (PSC) activation, invasion, and migration. (a, b) PSCs were treated with increasing doses of RSV (0, 12.5, 25, 50, 100, and 200 *μ*M) for 24 h, 48 h, and 72 h or H_2_O_2_ (0, 12.5, 25, 37.5, 50, 62.5, 75, 87.5, 100, 150, 200, and 300 *μ*M) for 24 h and then subjected to MTT assays to evaluate cell viability. (c–f) PSCs were treated with 50 *μ*M RSV or 10 mM N-acetyl-L-cysteine (NAC), a ROS scavenger, for 24 h prior to the 24 h incubation with 50 *μ*M H_2_O_2_. Protein levels of *α*-SMA were analyzed using Western blot and immunofluorescence staining of *α*-SMA. The red signal represents *α*-SMA staining, and nuclear DNA staining by DAPI is shown in blue (magnification, ×200; scale bar: 50 *μ*m). (g, h) PSCs were treated in groups as indicated, and migratory ability was determined by wound healing assays. The relative distance moved by the leading edge marked by black lines was measured 24 h after wounding with a sterile pipette tip (magnification, ×100; scale bar: 100 *μ*m). (i, j) PSCs were treated in groups as indicated, and invasive ability was determined by Matrigel invasion assays (magnification, ×200; scale bar: 50 *μ*m). Column: mean; bar: SD; ^∗^ *p* < 0.05 compared with the control group; ^#^ *p* < 0.05 compared with the H_2_O_2_ group.

PSCs were treated with increasing doses of RSV (0, 12.5, 25, 50, 100, and 200 *μ*M) for 24 h, 48 h, and 72 h and then subjected to MTT assays to evaluate cell viability (Figure 1(a)). RSV inhibited PSC proliferation in a dose- and time-dependent manner. Low concentrations of RSV (12.5, 25, and 50 *μ*M) exhibited little cytotoxicity, but treatment for longer periods (48 and 72 h) and at high concentrations (100 and 200 *μ*M) substantially decreased cell viability. Exogenous H_2_O_2_, a traditional cellular ROS inducer, exhibited growth-promoting action up to a concentration of approximately 50 *μ*M (Figure 1(b)). Therefore, we chose 50 *μ*M RSV and 50 *μ*M H_2_O_2_ for subsequent experiments. Oil Red O staining showed that 50 *μ*M H_2_O_2_ efficiently transferred the quiescent state of PSCs to the activated state with a considerable decline of lipid droplets (Figure S1). As shown in Figure S2, RSV at 50 *μ*M hardly exerted function on the protein expression of *α*-SMA and glycolytic enzymes in quiescent PSCs, so we focused on the potential functions of RSV when PSCs were activated. Next, to evaluate the effects of RSV and ROS on PSC activation, we treated PSCs with 50 *μ*M RSV or 10 mM NAC (a ROS scavenger) for 24 h prior to the 24 h incubation with 50 *μ*M H_2_O_2_. The protein expression of *α*-SMA was analyzed by Western blot (Figures 1(c) and 1(e)), and immunofluorescence staining of *α*-SMA was performed (Figures 1(d) and 1(f)). The expression of *α*-SMA was increased after H_2_O_2_ incubation, whereas NAC or RSV reversed this effect, indicating that H_2_O_2_-driven ROS-promoted PSC activation could be inhibited by RSV. To explore whether the migratory or invasive ability of PSCs is affected by ROS or RSV, wound healing assays and Matrigel invasion assays were performed. As shown in Figures 1(g) and 1(h), cell migratory ability was enhanced by H_2_O_2_ incubation, whereas NAC and RSV efficiently repressed this effect. The number of cells that invaded into the lower chamber was also increased by H_2_O_2_ stimulation but markedly decreased when the cells were pretreated with NAC or RSV (Figures 1(i) and 1(j)). Together, these results suggest that RSV potently inhibits H_2_O_2_-promoted PSC activation, invasion, and migration.

### 3.2. RSV Impedes H_2_O_2_-Driven ROS-Induced Glycolysis in PSCs

**Figure 2:**
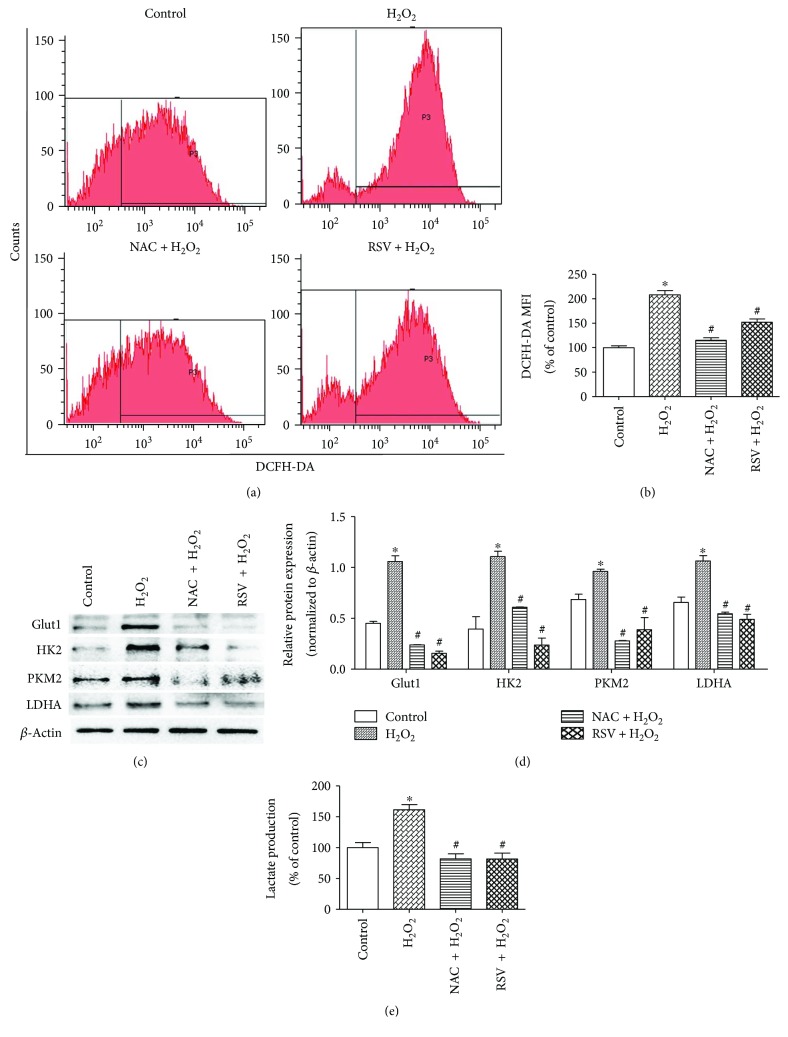
RSV impedes H_2_O_2_-driven ROS-induced glycolysis in PSCs. (a, b) PSCs were treated in groups as indicated, and ROS were detected using DCFH-DA probes. Representative flow cytometric images and the mean fluorescence intensity (MFI) of each group are shown. (c, d) PSCs from indicated groups were extracted to detect Glut1, HK2, PKM2, and LDHA levels by Western blot, (e) and the culture media (CM) were collected to measure lactate production. Lactate production was normalized by the concentration of protein in each group. Column: mean; bar: SD; ^∗^ *p* < 0.05 compared with the control group; ^#^ *p* < 0.05 compared with the H_2_O_2_ group.

Intracellular ROS levels were detected using DCFH-DA probes. As shown in Figures 2(a) and 2(b), H_2_O_2_-induced ROS upregulation was downregulated by NAC and repressed by RSV. To assess whether ROS or RSV affects glycolysis in PSCs, several pivotal glycolytic enzymes were assayed. As shown in Figures 2(c) and 2(d), glucose transporter 1 (Glut1), hexokinase 2 (HK2), pyruvate kinase M2 (PKM2), and lactate dehydrogenase A (LDHA) levels were elevated under ROS treatment but decreased if cells were pretreated with NAC or RSV. Similarly, the enhancement by ROS of the production of lactate, an important metabolite transferred from PSCs to “fertilize” neighboring cancer cells [32], was hindered by pretreatment with NAC or RSV (Figure 2(e)). These results demonstrate that RSV can inhibit H_2_O_2_-driven ROS-induced glycolysis of PSCs.

### 3.3. RSV Reduces ROS-Induced miR-21 Expression and Increases PTEN Expression in PSCs

**Figure 3:**
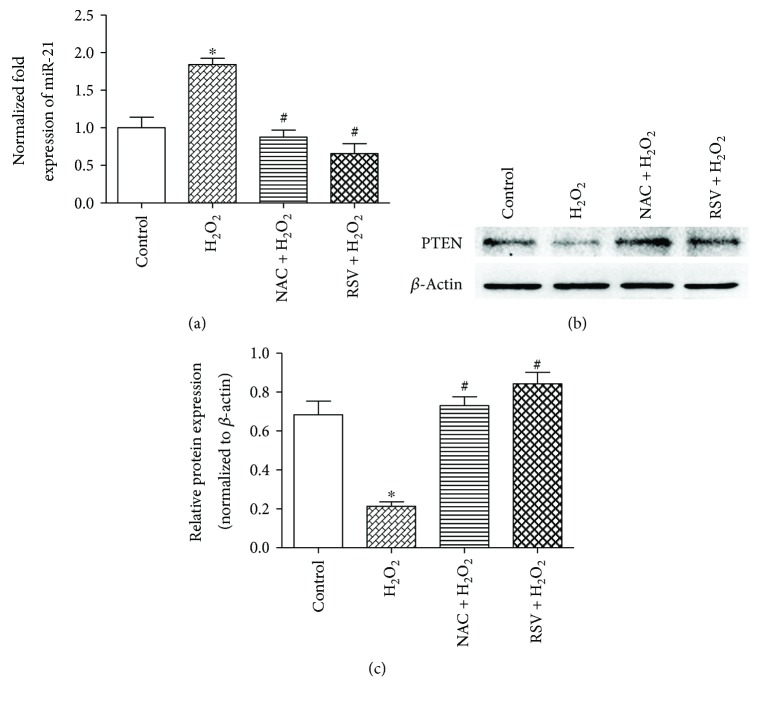
RSV reduces ROS-induced miR-21 expression and increases PTEN expression in PSCs. (a) PSCs were treated in groups as indicated, and qRT-PCR analysis was performed to detect miR-21 expression. (b, c) Cells were treated in groups as indicated, and the protein level of PTEN was detected by Western blot. Column: mean; bar: SD; ^∗^ *p* < 0.05 compared with the control group; ^#^ *p* < 0.05 compared with the H_2_O_2_ group.

miR-21 levels are reportedly upregulated by H_2_O_2_ treatment in cardiac myocytes [33], and RSV inhibits miR-21 expression in several types of cancer cells [34–36]. However, whether the level of miR-21 and its target genes in PSCs are regulated by RSV or H_2_O_2_ is unknown. Our results showed that PSCs treated with H_2_O_2_ displayed higher levels of miR-21, and this enhancement was reversed by NAC or RSV (Figure 3(a)). Moreover, RSV and NAC restored PTEN expression, which was downregulated by H_2_O_2_ (Figures 3(b) and 3(c)).

### 3.4. miR-21 Downregulation Attenuates ROS-Induced Activation, Migration, Invasion, and Glycolysis of PSCs

**Figure 4:**
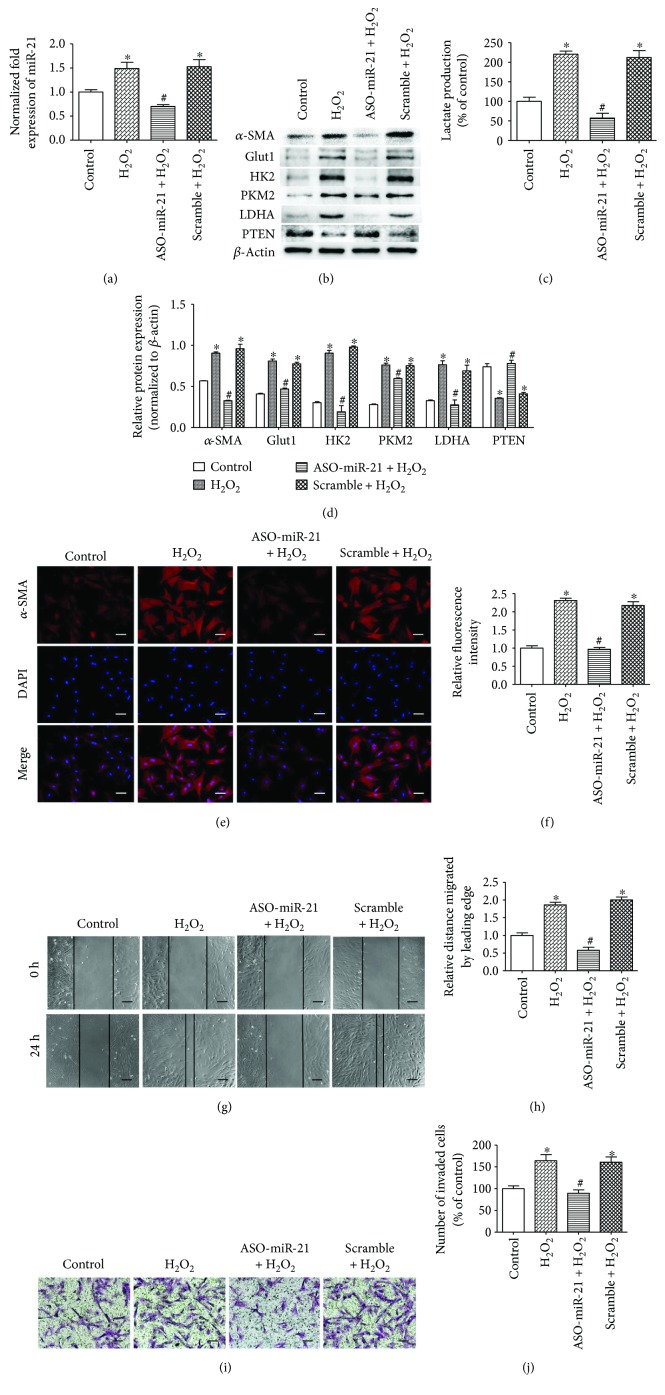
miR-21 downregulation attenuates the ROS-induced activation, migration, and glycolysis of PSCs. (a) PSCs were transfected with the miR-21 inhibitor (100 nM) or scrambled inhibitor (100 nM) 24 h prior to the 24 h incubation with 50 *μ*M H_2_O_2_, and RT-PCR analysis was performed to detect miR-21 expression. (b–d) PSCs from indicated groups were extracted to detect *α*-SMA, Glut1, HK2, PKM2, LDHA, and PTEN levels by Western blot, and the CM were collected to measure lactate production. Lactate production was normalized by the concentration of protein in each group. (e, f) Immunofluorescence staining of *α*-SMA was performed. The red signal represents *α*-SMA staining, and nuclear DNA staining by DAPI is shown in blue (magnification, ×200; scale bar: 50 *μ*m). (g, h) PSCs were treated in groups as indicated, and migratory ability was determined by wound healing assays. The relative distance moved by the leading edge marked by black lines was measured 24 h after wounding with a sterile pipette tip (magnification, ×100; scale bar: 100 *μ*m). (i, j) PSCs were treated in groups as indicated, and invasive ability was determined by Matrigel invasion assays (magnification, ×200; scale bar: 50 *μ*m). Column: mean; bar: SD; ^∗^ *p* < 0.05 compared with the control group; ^#^ *p* < 0.05 compared with the scramble + H_2_O_2_ group.

To evaluate the role of miR-21 in the ROS-induced properties of PSCs, loss-of-function analysis using a specific antisense oligonucleotide targeting miR-21 was performed. The miR-21 inhibitor effectively repressed the expression of the microRNA (Figure 4(a)) and enhanced PTEN levels (Figure 4(b)). As shown in Figures 4(b)–4(f), miR-21 inhibited oxidative stress-promoted glycolysis, as represented by Glut1, HK2, PKM2, and LDHA expression and lactate production, and impeded PSC activation, as evidenced by the downregulated protein expression and fluorescence intensity of *α*-SMA. Our results further demonstrated that the enhancement of cell migratory ability by H_2_O_2_ incubation was reduced by miR-21 inhibitor pretreatment (Figures 4(g) and 4(h). The number of cells that invaded into the lower chamber also decreased with miR-21 pretreatment, despite H_2_O_2_ stimulation (Figures 4(i) and 4(j)). Taken together, these results suggest that miR-21 plays a vital part in the ROS-induced activation, migration, invasion, and glycolysis of PSCs.

### 3.5. miR-21 Is Essential for RSV-Induced Responses of PSCs

**Figure 5:**
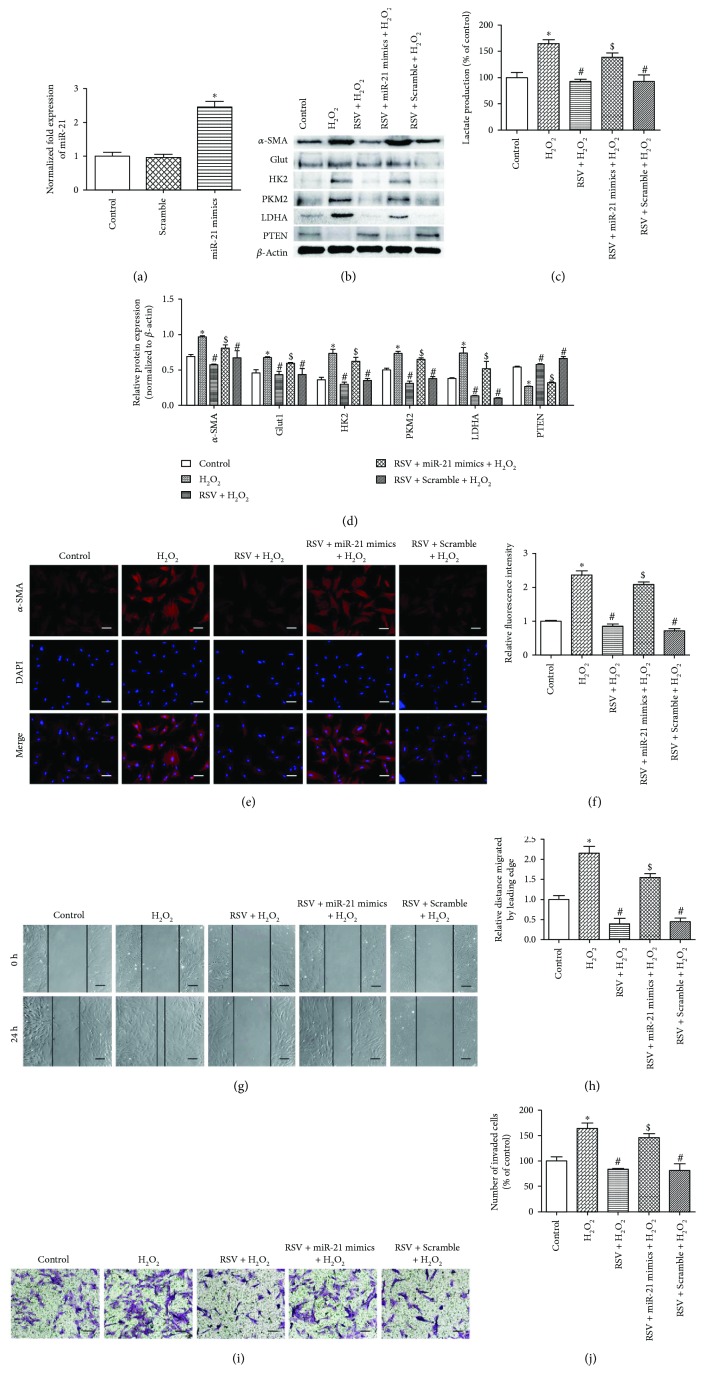
miR-21 is essential for RSV-mediated responses of PSCs. (a) PSCs were transfected with miR-21 mimics (20 nM) or scrambled mimics (20 nM) for 24 h, and RT-PCR analysis was performed to detect miR-21 expression. (b–d) PSCs were treated with 50 *μ*M RSV for 24 h and then transfected with miR-21 mimics (20 nM) or scrambled mimics (20 nM) prior to the 24 h incubation with H_2_O_2_ at 50 *μ*M. PSCs from indicated groups were extracted to detect *α*-SMA, Glut1, HK2, PKM2, LDHA, and PTEN levels by Western blot, and the CM were collected to measure lactate production. Lactate production was normalized by the concentration of protein in each group. (e, f) Immunofluorescence staining of *α*-SMA was performed. The red signal represents *α*-SMA staining, and nuclear DNA staining by DAPI is shown in blue (magnification, ×200; scale bar: 50 *μ*m). (g, h) PSCs were treated in groups as indicated, and migratory ability was determined by wound healing assays. The relative distance moved by the leading edge marked by black lines was measured 24 h after wounding with a sterile pipette tip (magnification, ×100; scale bar: 100 *μ*m). (i, j) PSCs were treated in groups as indicated, and invasive ability was determined by Matrigel invasion assays (magnification, ×200; scale bar: 50 *μ*m). Column: mean; bar: SD; ^∗^ *p* < 0.05 compared with the control group; ^#^ *p* < 0.05 compared with the H_2_O_2_ group; ^$^ *p* < 0.05 compared with the RSV + scramble + H_2_O_2_ group.

To further examine whether miR-21 mediates the RSV-induced responses of PSCs, PSCs were treated with RSV for 24 h and then transfected with miR-21 mimics or scrambled mimics (Figure 5(a)) prior to incubation with H_2_O_2_. As shown in Figures 5(b) and 5(d), the expression levels of the activation marker *α*-SMA and the glycolytic enzymes Glut1, HK2, PKM2, and LDHA were rescued by the miR-21 mimics after RSV treatment. Fluorescent images of *α*-SMA staining (Figures 5(e) and 5(f)) and measurement of lactate production (Figure 5(c)) further confirmed these findings. Additionally, miR-21 mimics partly reversed the suppression by RSV of the migratory abilities (Figures 5(g) and 5(h)) and invasive activities (Figures 5(i) and 5(j)) of PSCs. In conclusion, our results indicate that miR-21 plays a pivotal role in mediating the reduction of the activation, migration, invasion, and glycolysis of PSCs by RSV.

### 3.6. RSV Inhibits Pancreatic Cancer Cell Invasion and Migration through Suppression of ROS/miR-21 in PSCs

**Figure 6:**
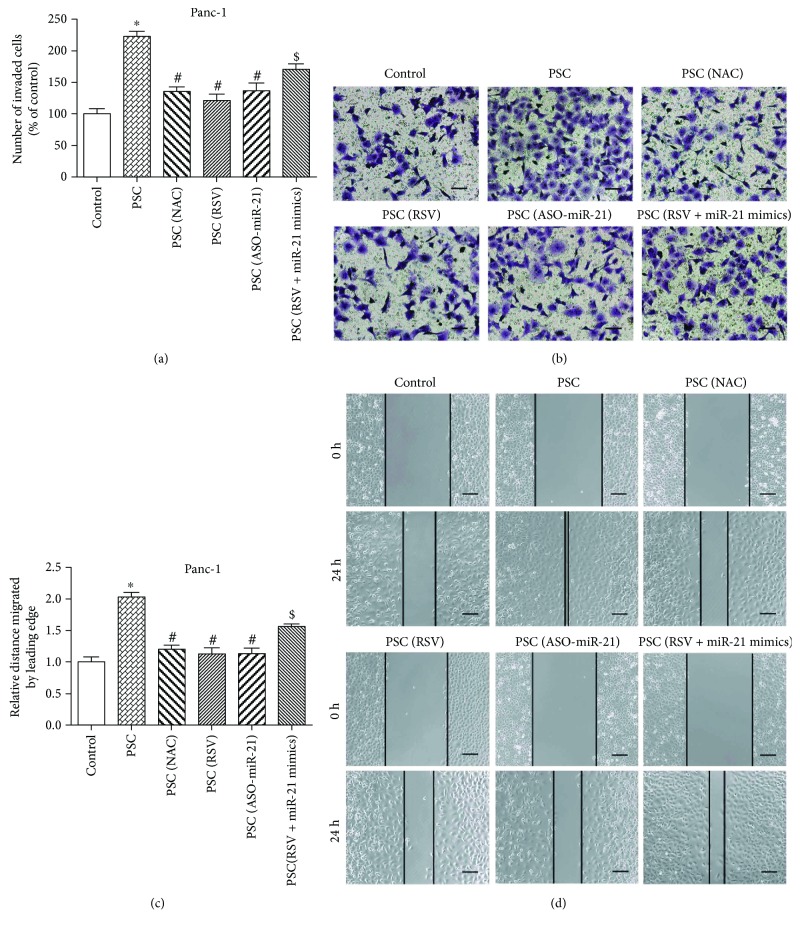
RSV inhibits pancreatic cancer cell invasion and migration through suppression of ROS/miR-21 in PSCs. (a, b) PSCs were treated in groups as indicated and then cocultured with Panc-1 cells using Transwell chambers. The invasive ability of Panc-1 cells was determined by Matrigel invasion assays (magnification, ×200; scale bar: 50 *μ*m). (c, d) PSCs were treated in groups as indicated and then cocultured with Panc-1 cells using Millicell hanging cell culture inserts. The migratory ability of Panc-1 cells was determined by wound healing assays. The relative distance moved by the leading edge marked by black lines was measured 24 h after wounding with a sterile pipette tip (magnification, ×100; scale bar: 100 *μ*m). Column: mean; bar: SD; ^∗^ *p* < 0.05 compared with the control group; ^#^ *p* < 0.05 compared with the PSC group; ^$^ *p* < 0.05 compared with the PSC (RSV) group.

Many factors secreted from PSCs, such as cytokines, growth factors, neurotrophic factors, and chemotactic factors, mediate the proinvasion and promigration abilities of PCCs [5]. Moreover, lactate secretion from PSCs is essential for accelerating tumor growth [12, 32]. Our results above demonstrated that lactate production is upregulated upon oxidative stress but reduced upon NAC treatment and that miR-21 mediates the reduction of lactate secretion by RSV. To assess whether RSV or NAC administration and miR-21 expression in PSCs modulate the invasion or migration of PCCs, we tested Panc-1 tumor cells cocultured with different groups of PSCs. Both the invasive ability (Figures 6(a) and 6(b)) and migratory activity (Figures 6(c) and 6(d)) of Panc-1 cells were enhanced when cocultured with PSCs, and these increases were mostly abrogated when PSCs were pretreated with NAC, RSV, or miR-21 inhibitor. Incubation of PSCs with miR-21 mimics partly reversed the repression of the invasive ability and migratory activity of cocultured Panc-1 cells by RSV treatment.

## 4. Discussion

Despite extensive study and the elucidation of the underlying mechanisms in greater detail, PDAC remains a notoriously malignant tumor characterized by the lack of an effective therapeutic strategy and poor life expectancy. Gemcitabine, the standard chemotherapeutic agent for PDAC, is indispensable but exhibits restricted effects [37], and combination chemotherapy regimens such as FOLFIRINOX, which is composed of folinic acid, 5-fluorouracil (5-FU), irinotecan, and oxaliplatin, show efficacy but also toxicity [38, 39]. Novel chemotherapeutic agents and therapeutic targets are urgently required.

RSV, a natural polyphenolic phytoalexin found in the skins of grapes and other berries and some Chinese medicines, has extensive functions in tumor therapies. RSV inhibits proliferation, induces apoptosis, represses invasion and migration, and impairs tumor-initiating stem-like properties via several signaling pathways, such as the sonic hedgehog pathway [40, 41] and the PI3K/Akt/NF-*κ*B pathway [42]. Together with these classical regulatory pathways, RSV has been shown to regulate the expression of microRNAs (miRNAs) by which RSV may exert anti-inflammation and antitumor effects [28]. An in vivo study has explored the function of RSV on modulating the expression of miRNAs in an ischemia/reperfusion model of rat, and over 25 miRNAs were observed [43]. It was shown in breast cancer cells that miR-125b-5p, miR-200c-3p, miR-409-3p, miR-122-5p, and miR-542-3p were modulated by RSV, thereby affecting antiapoptotic and cell cycle proteins such as Bcl-2 and CDKs [44]. Using microarray approaches, 51 miRNAs were also found to be regulated by RSV in prostate cancer [45]. Combinations of single-agent treatments occasionally show synergetic effects. As food complements, RSV together with capsaicin enhances the effect of gemcitabine [23], and we previously demonstrated that RSV enhances the sensitivity of PCCs to gemcitabine [25]. However, the antitumor mechanisms of RSV warrant further study.

The desmoplastic stroma plays a critical role in tumor growth and aggression, and PSCs are at the center of this progression. When PSCs compose the cellular population of a PCC and PSC admixture at a high fraction of 0.66–0.83, a maximal effect on promoting cancer cell proliferation and invasion is observed [46]. PSCs are aberrantly activated and transformed into cancer-associated fibroblasts, which facilitate tumor malignancy. Cancer cells can secrete H_2_O_2_, which triggers oxidative stress in adjacent PSCs [8], and oxidative stress is essential in promoting PSC activation [47], and our data showed that H_2_O_2_-induced ROS promoted the expression of *α*-SMA in PSCs. However, Kikuta et al. [48] reported that the transformation of freshly isolated PSCs into the activated phenotype is not initiated by H_2_O_2_; this discrepancy may be explained by differences in culture conditions and the potential for further promotion of PSC activation by H_2_O_2_-induced ROS once the PSCs were preactivated in culture. All experiments were performed using human-derived PSCs in our research, and the cells were incubated with 50 *μ*M H_2_O_2_ for 24 h, whereas Kikuta et al. treated freshly isolated rat PSCs with 100 *μ*M H_2_O_2_ for 7 days. It was demonstrated that *trans*-resveratrol possesses potent antifibrotic activities as it suppressed TGF-beta-induced PSC activation [49]. Our results also showed that resveratrol served as a potential therapeutic agent in antifibrotic approaches by reducing ROS-induced PSC activation. In addition, RSV and NAC inhibited the migration and invasion of PSCs, two important characteristics of activated PSCs [50].

Cancer-associated fibroblasts such as PSCs usually undergo oxidative stress, mitophagy, and autophagy and exhibit glycolytic phenotypes after “fertilization” by tumor cells. These fibroblasts then “back-nurture” adjacent cancer cells by secreting lactate, ketone, and other metabolites [11, 12, 51]. This two-compartment theory is a great addition to the traditional “Warburg effect” in tumor cells and may provide new insights on the crucial role of the microenvironment in tumor development. RSV can impede glycolysis in tumor cells [29, 30], but its effects on the glycolytic phenotypes of PSCs have not been explored. Here, we found that H_2_O_2_-induced ROS promoted glycolysis in PSCs, whereas RSV downregulated the expression of key glycolytic enzymes and, more importantly, decreased lactate production.

As an oncomiR or oncogenic miRNA, miR-21 is abundantly expressed in several types of cancers [13, 15, 34]. It has been reported that miR-21 and CCN2 compose a positive feedback loop during PSC activation [52] and that inhibition of miR-21 leads to decreased migration and invasion of PSCs [53]. In the present study, we found that miR-21 was necessary for ROS-promoted PSC activation, and miR-21 downregulation disrupted the ROS-promoted migration and invasion of PSCs. Previous studies have demonstrated that miR-21 is critical in maintaining the “Warburg effect” in bladder cancer cells [20] and can enhance glycolysis in nonsmall cell lung cancer cells, which might contribute to radiation resistance [54]. Accordingly, we explored the role of miR-21 in modulating glycolysis in PSCs and found that miR-21 was an essential component of the promotion of glycolysis in PSCs. Notably, AKT is regarded as a “Warburg kinase,” and mTOR links tumor cell metabolism and oncogenic signaling [55, 56]. Accordingly, as a downstream pathway of miR-21, the PTEN/PI3K/AKT/mTOR pathway might exhibit several functions modulating cell metabolism whose mechanisms require further elucidation. Recent studies have suggested that RSV might function by affecting miR-21 in prostate cancer cells [34] and PCCs [35]. This connection was further demonstrated in stroma, in which our results showed that miR-21 partly mediated the responses of PSCs to RSV.

Accumulating evidence indicates that the interaction between tumor and stroma is vital in tumor progression and that the underlying mechanisms are complicated. PCCs secrete stimulants such as TGF-*β*1, PDGF, SHH, and H_2_O_2_ to the microenvironment to activate the transformation of PSCs into cancer-associated myofibroblasts. In turn, activated PSCs release nutrients such as cytokines, chemokines, growth factors, and extracellular matrix proteins to further support tumor cells. It is worth noting that the lactate shuttle is an important part of the tumor-stroma interplay [32, 57]. Activated PSCs undergo metabolic reprogramming toward a Warburg phenotype, and lactate is secreted through monocarboxylate transporter 4 (MCT4) to be uploaded by monocarboxylate transporter 1 (MCT1) expressed in tumor cells for tumor growth. In the present study, we found that lactate production from PSCs was upregulated by oxidative stress but reduced by NAC treatment, and miR-21 mediated RSV-impeded lactate secretion. In line with the above findings on lactate, the enhanced invasive and migratory activities of Panc-1 cells cocultured with PSCs were mostly abrogated when PSCs were pretreated with a NAC, RSV, or miR-21 inhibitor, and RSV might function partly through modulating miR-21 in PSCs. However, direct regulation of lactate and other possible soluble factors, such as PDGF, SDF-1, and IL-6, was not assayed in the present study, which points the way for further investigation of detailed mechanisms involved in the tumor-stroma interaction.

## 5. Conclusions

To the best of our knowledge, this is the first study to report that miR-21 acts as a molecular switch in the “reverse Warburg effect” in PSCs and that RSV inhibits ROS-promoted PSC migration, invasion, and glycolysis, and miR-21 mediates these responses to RSV in PSCs. More importantly, RSV administration or suppression of ROS/miR-21 in PSCs ameliorates the invasive and migratory abilities of PCCs under coculture, suggesting that in addition to inhibiting the invasion and migration of PCCs directly, RSV may function by affecting PSCs or the interplay between tumor and stroma.

## Supplementary Materials

Supplementary Materials Table S1: the sequences of the miRNA inhibitor, mimics, and control. Figure S1: H_2_O_2_ transfers quiescent PSCs to the activated state determined by Oil Red O staining. Scale bar: 50 *μ*m. Figure S2: RSV hardly affects protein expression of *α*-SMA and glycolytic enzymes in quiescent PSCs. (A) Quiescent PSCs were treated with 50 *μ*M RSV for 24 h. Proteins from indicated groups were extracted to detect *α*-SMA, Glut1, HK2, PKM2, and LDHA levels by Western blot, and (B) the relative protein expression was normalized by *β*-actin in each group.

## References

[1] Siegel R. L., Miller K. D., Jemal A. (2017). Cancer statistics, 2017. *CA: A Cancer Journal for Clinicians*.

[2] Rahib L., Smith B. D., Aizenberg R., Rosenzweig A. B., Fleshman J. M., Matrisian L. M. (2014). Projecting cancer incidence and deaths to 2030: the unexpected burden of thyroid, liver, and pancreas cancers in the United States. *Cancer Research*.

[3] von Ahrens D., Bhagat T. D., Nagrath D., Maitra A., Verma A. (2017). The role of stromal cancer-associated fibroblasts in pancreatic cancer. *Journal of Hematology & Oncology*.

[4] Nielsen M. F. B., Mortensen M. B., Detlefsen S. (2016). Key players in pancreatic cancer-stroma interaction: cancer-associated fibroblasts, endothelial and inflammatory cells. *World Journal of Gastroenterology*.

[5] Haqq J., Howells L. M., Garcea G., Metcalfe M. S., Steward W. P., Dennison A. R. (2014). Pancreatic stellate cells and pancreas cancer: current perspectives and future strategies. *European Journal of Cancer*.

[6] Masamune A., Watanabe T., Kikuta K., Shimosegawa T. (2009). Roles of pancreatic stellate cells in pancreatic inflammation and fibrosis. *Clinical Gastroenterology and Hepatology*.

[7] Tang D., Wang D., Yuan Z. (2013). Persistent activation of pancreatic stellate cells creates a microenvironment favorable for the malignant behavior of pancreatic ductal adenocarcinoma. *International Journal of Cancer*.

[8] Martinez-Outschoorn U. E., Lin Z., Trimmer C. (2011). Cancer cells metabolically “fertilize” the tumor microenvironment with hydrogen peroxide, driving the Warburg effect: Implications for PET imaging of human tumors. *Cell Cycle*.

[9] Lei J., Huo X., Duan W. (2014). *α*-Mangostin inhibits hypoxia-driven ROS-induced PSC activation and pancreatic cancer cell invasion. *Cancer Letters*.

[10] Wehr A. Y., Furth E. E., Sangar V., Blair I. A., Yu K. H. (2011). Analysis of the human pancreatic stellate cell secreted proteome. *Pancreas*.

[11] Pavlides S., Whitaker-Menezes D., Castello-Cros R. (2009). The reverse Warburg effect: aerobic glycolysis in cancer associated fibroblasts and the tumor stroma. *Cell Cycle*.

[12] Pavlides S., Vera I., Gandara R. (2012). Warburg meets autophagy: cancer-associated fibroblasts accelerate tumor growth and metastasis via oxidative stress, mitophagy, and aerobic glycolysis. *Antioxidants & Redox Signaling*.

[13] Sicard F., Gayral M., Lulka H., Buscail L., Cordelier P. (2013). Targeting miR-21 for the therapy of pancreatic cancer. *Molecular Therapy*.

[14] Peralta-Zaragoza O., Deas J., Meneses-Acosta A. (2016). Relevance of miR-21 in regulation of tumor suppressor gene PTEN in human cervical cancer cells. *BMC Cancer*.

[15] Wickramasinghe N. S., Manavalan T. T., Dougherty S. M., Riggs K. A., Li Y., Klinge C. M. (2009). Estradiol downregulates miR-21 expression and increases miR-21 target gene expression in MCF-7 breast cancer cells. *Nucleic Acids Research*.

[16] Kadera B. E., Li L., Toste P. A. (2013). MicroRNA-21 in pancreatic ductal adenocarcinoma tumor-associated fibroblasts promotes metastasis. *PLoS One*.

[17] Uozaki H., Morita S., Kumagai A. (2014). Stromal miR-21 is more important than miR-21 of tumour cells for the progression of gastric cancer. *Histopathology*.

[18] Mitra A. K., Zillhardt M., Hua Y. (2012). MicroRNAs reprogram normal fibroblasts into cancer-associated fibroblasts in ovarian cancer. *Cancer Discovery*.

[19] Park J. K., Lee E. J., Esau C., Schmittgen T. D. (2009). Antisense inhibition of microRNA-21 or -221 arrests cell cycle, induces apoptosis, and sensitizes the effects of gemcitabine in pancreatic adenocarcinoma. *Pancreas*.

[20] Yang X., Cheng Y., Li P. (2015). A lentiviral sponge for miRNA-21 diminishes aerobic glycolysis in bladder cancer T24 cells via the PTEN/PI3K/AKT/mTOR axis. *Tumour Biology*.

[21] Jagdeo J., Adams L., Lev-Tov H., Sieminska J., Michl J., Brody N. (2010). Dose-dependent antioxidant function of resveratrol demonstrated via modulation of reactive oxygen species in normal human skin fibroblasts in vitro. *Journal of Drugs in Dermatology*.

[22] Savi M., Bocchi L., Sala R. (2016). Parenchymal and stromal cells contribute to pro-inflammatory myocardial environment at early stages of diabetes: protective role of resveratrol. *Nutrients*.

[23] Vendrely V., Peuchant E., Buscail E. (2017). Resveratrol and capsaicin used together as food complements reduce tumor growth and rescue full efficiency of low dose gemcitabine in a pancreatic cancer model. *Cancer Letters*.

[24] Karimi Dermani F., Saidijam M., Amini R., Mahdavinezhad A., Heydari K., Najafi R. (2017). Resveratrol inhibits proliferation, invasion, and epithelial–mesenchymal transition by increasing miR-200c expression in HCT-116 colorectal cancer cells. *Journal of Cellular Biochemistry*.

[25] Jiang Z., Chen X., Chen K. (2016). YAP inhibition by resveratrol via activation of AMPK enhances the sensitivity of pancreatic cancer cells to gemcitabine. *Nutrients*.

[26] Roy S. K., Chen Q., Fu J., Shankar S., Srivastava R. K. (2011). Resveratrol inhibits growth of orthotopic pancreatic tumors through activation of FOXO transcription factors. *PLoS One*.

[27] Rauf A., Imran M., Suleria H. A. R., Ahmad B., Peters D. G., Mubarak M. S. (2017). A comprehensive review of the health perspectives of resveratrol. *Food & Function*.

[28] Kumar A., Rimando A. M., Levenson A. S. (2017). Resveratrol and pterostilbene as a microRNA-mediated chemopreventive and therapeutic strategy in prostate cancer. *Annals of the New York Academy of Sciences*.

[29] Li W., Ma X., Li N. (2016). Resveratrol inhibits hexokinases II mediated glycolysis in non-small cell lung cancer via targeting Akt signaling pathway. *Experimental Cell Research*.

[30] Gomez L. S., Zancan P., Marcondes M. C. (2013). Resveratrol decreases breast cancer cell viability and glucose metabolism by inhibiting 6-phosphofructo-1-kinase. *Biochimie*.

[31] Gao Z., Wang X., Wu K., Zhao Y., Hu G. (2010). Pancreatic stellate cells increase the invasion of human pancreatic cancer cells through the stromal cell-derived Factor-1/CXCR4 axis. *Pancreatology*.

[32] Whitaker-Menezes D., Martinez-Outschoorn U. E., Lin Z. (2011). Evidence for a stromal-epithelial “lactate shuttle” in human tumors: MCT4 is a marker of oxidative stress in cancer-associated fibroblasts. *Cell Cycle*.

[33] Cheng Y., Liu X., Zhang S., Lin Y., Yang J., Zhang C. (2009). MicroRNA-21 protects against the H(2)O(2)-induced injury on cardiac myocytes via its target gene PDCD4. *Journal of Molecular and Cellular Cardiology*.

[34] Sheth S., Jajoo S., Kaur T. (2012). Resveratrol reduces prostate cancer growth and metastasis by inhibiting the Akt/MicroRNA-21 pathway. *PLoS One*.

[35] Liu P., Liang H., Xia Q. (2013). Resveratrol induces apoptosis of pancreatic cancers cells by inhibiting miR-21 regulation of BCL-2 expression. *Clinical & Translational Oncology*.

[36] Wang G., Dai F., Yu K. (2015). Resveratrol inhibits glioma cell growth via targeting oncogenic microRNAs and multiple signaling pathways. *International Journal of Oncology*.

[37] Oettle H., Post S., Neuhaus P. (2007). Adjuvant chemotherapy with gemcitabine vs observation in patients undergoing curative-intent resection of pancreatic cancer: a randomized controlled trial. *JAMA*.

[38] Lee J.-c., Kim J. W., Ahn S. (2017). Optimal dose reduction of FOLFIRINOX for preserving tumour response in advanced pancreatic cancer: using cumulative relative dose intensity. *European Journal of Cancer*.

[39] Baldini C., Escande A., Bouché O. (2017). Safety and efficacy of FOLFIRINOX in elderly patients with metastatic or locally advanced pancreatic adenocarcinoma: a retrospective analysis. *Pancreatology*.

[40] Mo W., Xu X., Xu L. (2011). Resveratrol inhibits proliferation and induces apoptosis through the hedgehog signaling pathway in pancreatic cancer cell. *Pancreatology*.

[41] Li W., Cao L., Chen X., Lei J., Ma Q. (2016). Resveratrol inhibits hypoxia-driven ROS-induced invasive and migratory ability of pancreatic cancer cells via suppression of the Hedgehog signaling pathway. *Oncology Reports*.

[42] Li W., Ma J., Ma Q. (2013). Resveratrol inhibits the epithelial-mesenchymal transition of pancreatic cancer cells via suppression of the PI-3K/Akt/NF-*κ*B Pathway. *Current Medicinal Chemistry*.

[43] Mukhopadhyay P., Mukherjee S., Ahsan K., Bagchi A., Pacher P., Das D. K. (2010). Restoration of altered microRNA expression in the ischemic heart with resveratrol. *PLoS One*.

[44] Venkatadri R., Muni T., Iyer A. K. V., Yakisich J. S., Azad N. (2016). Role of apoptosis-related miRNAs in resveratrol-induced breast cancer cell death. *Cell Death & Disease*.

[45] Dhar S., Hicks C., Levenson A. S. (2011). Resveratrol and prostate cancer: promising role for microRNAs. *Molecular Nutrition & Food Research*.

[46] Kadaba R., Birke H., Wang J. (2013). Imbalance of desmoplastic stromal cell numbers drives aggressive cancer processes. *The Journal of Pathology*.

[47] Ryu G. R., Lee E., Chun H. J. (2013). Oxidative stress plays a role in high glucose-induced activation of pancreatic stellate cells. *Biochemical and Biophysical Research Communications*.

[48] Kikuta K., Masamune A., Satoh M., Suzuki N., Satoh K., Shimosegawa T. (2006). Hydrogen peroxide activates activator protein-1 and mitogen-activated protein kinases in pancreatic stellate cells. *Molecular and Cellular Biochemistry*.

[49] Tsang S. W., Zhang H., Lin Z., Mu H., Bian Z. X. (2015). Anti-fibrotic effect of trans-resveratrol on pancreatic stellate cells. *Biomedicine & Pharmacotherapy*.

[50] Phillips P. A., Wu M. J., Kumar R. K. (2003). Cell migration: a novel aspect of pancreatic stellate cell biology. *Gut*.

[51] Capparelli C., Guido C., Whitaker-Menezes D. (2012). Autophagy and senescence in cancer-associated fibroblasts metabolically supports tumor growth and metastasis, via glycolysis and ketone production. *Cell Cycle*.

[52] Charrier A., Chen R., Chen L. (2014). Connective tissue growth factor (CCN2) and microRNA-21 are components of a positive feedback loop in pancreatic stellate cells (PSC) during chronic pancreatitis and are exported in PSC-derived exosomes. *Journal of Cell Communication and Signaling*.

[53] Ali S., Suresh R., Banerjee S. (2015). Contribution of microRNAs in understanding the pancreatic tumor microenvironment involving cancer associated stellate and fibroblast cells. *American Journal of Cancer Research*.

[54] Jiang S., Wang R., Yan H., Jin L., Dou X., Chen D. (2016). MicroRNA-21 modulates radiation resistance through upregulation of hypoxia-inducible factor-1*α*-promoted glycolysis in non-small cell lung cancer cells. *Molecular Medicine Reports*.

[55] Robey R. B., Hay N. (2009). Is Akt the “Warburg kinase”?—Akt-energy metabolism interactions and oncogenesis. *Seminars in Cancer Biology*.

[56] Zha X., Sun Q., Zhang H. (2011). mTOR upregulation of glycolytic enzymes promotes tumor development. *Cell Cycle*.

[57] Fiaschi T., Marini A., Giannoni E. (2012). Reciprocal metabolic reprogramming through lactate shuttle coordinately influences tumor-stroma interplay. *Cancer Research*.

