# Supplementary material for: Resveratrol Inhibits ROS-Promoted Activation and Glycolysis of Pancreatic Stellate Cells via Suppression of miR-21
Source: Oxid Med Cell Longev. 2018 Apr 26;2018:1346958. doi: 10.1155/2018/1346958 (PMC5944235; doi:10.1155/2018/1346958)
Supplement: Supplementary Materials — Table S1: the sequences of the miRNA inhibitor, mimics, and control. Figure S1: H2O2 transfers quiescent PSCs to the activated state determined by Oil Red O staining. Scale bar: 50 μm. Figure S2: RSV hardly affects protein expression of α-SMA and glycolytic enzymes in quiescent PSCs. (A) Quiescent PSCs were treated with 50 μM RSV for 24 h. Proteins from indicated groups were extracted to detect α-SMA, Glut1, HK2, PKM2, and LDHA levels by Western blot, and (B) the relative protein expression was normalized by β-actin in each group. [file 1346958.f1.docx]

***Supplementary Materials***

**Table S1.** The sequences of miRNA inhibitor, mimics and control.

| **Names** | **Primer Sequences** |
| --- | --- |
| miR-21 inhibitor | 5′-UCAACAUCAGUCUGAUAAGCUA-3′ |
| negative inhibitor | 5′-CAGUACUUUUGUGUAGUACAA-3′ |
| miR-21 mimics | sense: 5′-UAGCUUAUCAGACUGAUGUUGA-3′ |
|  | antisense: 5′-AACAUCAGUCUGAUAAGCUAUU-3′ |
| negative mimics | sense: 5′-UUCUUCGAACGUGUCACGUTT-3′ |
|  | antisense: 5′-ACGUGACACGUUCGGAGAATT-3′ |

**Fig.S1**


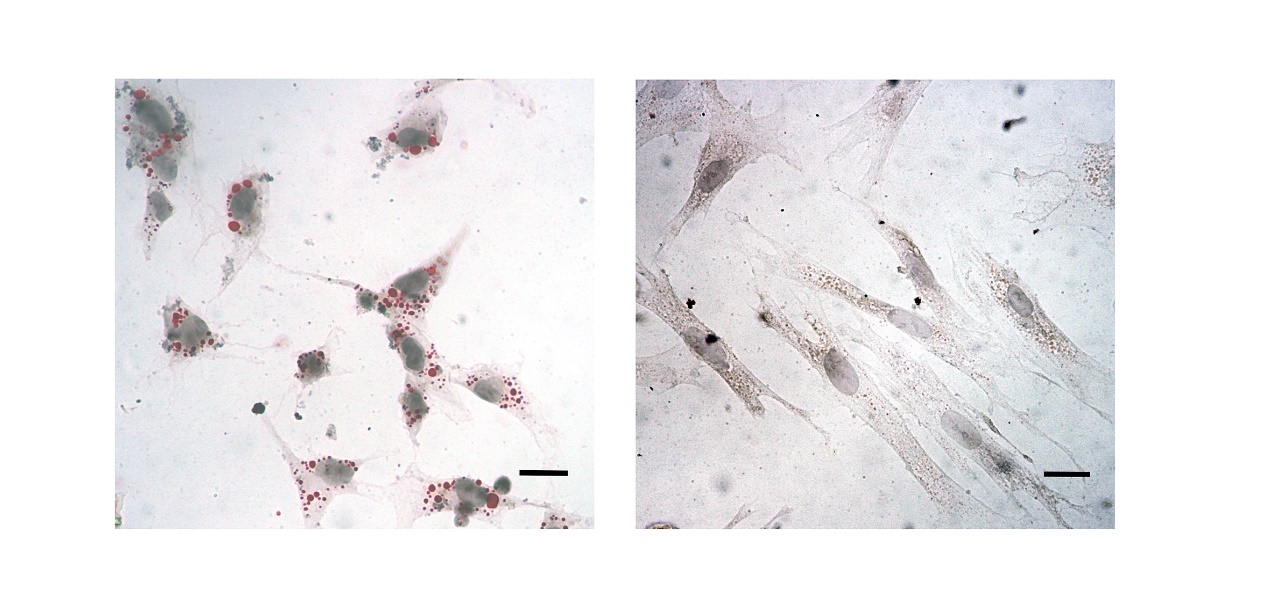


Fig.S1. H_2_O_2_ transfers quiescent PSCs to activated state determined by Oil red O staining. Scale bar: 50µm.

**Fig.S2**


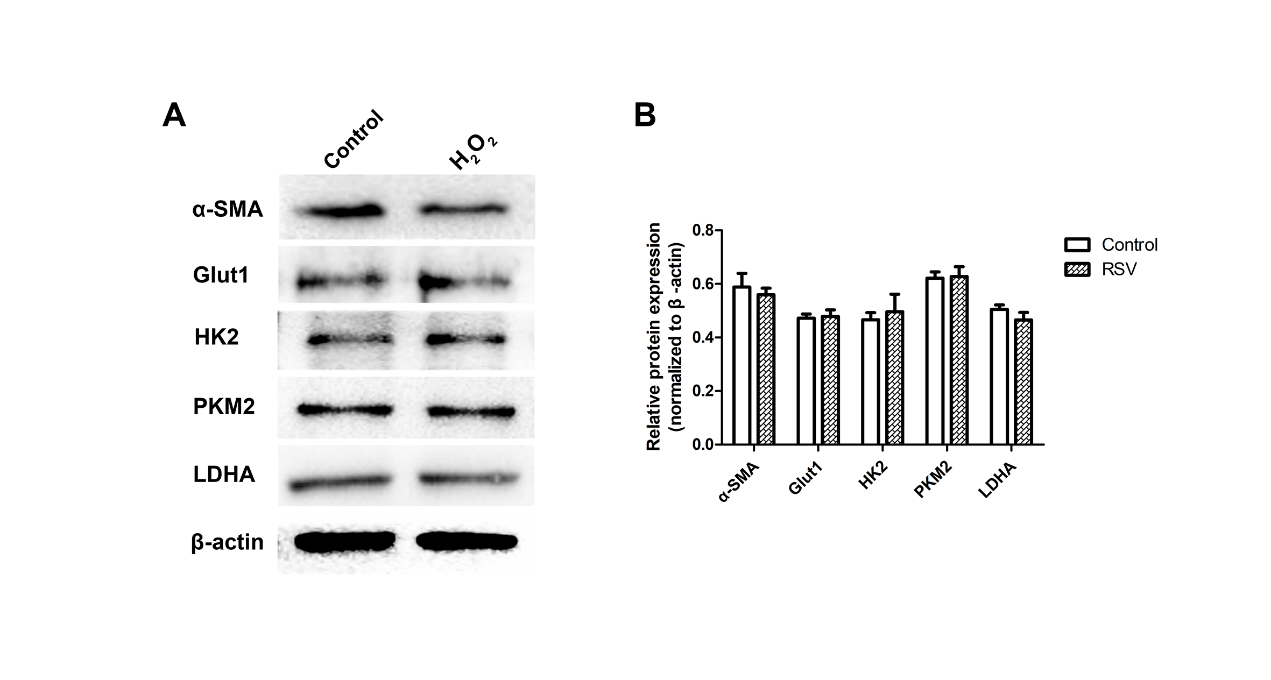


Figure S2. RSV hardly affects protein expression of α-SMA and glycolytic enzymes in quiescent PSCs. (A) Quiescent PSCs were treated with 50µM RSV for 24 h. Proteins from indicated groups were extracted to detect α-SMA, Glut1, HK2, PKM2, and LDHA levels by western blot, (B) and the relative protein expression was normalized by β-actin in each group.
